# Outcomes of adolescent and young patients with hepatocellular carcinoma after curative liver resection: a retrospective study

**DOI:** 10.1186/s12957-022-02658-3

**Published:** 2022-06-21

**Authors:** Zheng-Yun Zhang, Jiao Guan, Xin-Ping Wang, Di-Si Hao, Zun-Qiang Zhou

**Affiliations:** 1 Department of Surgery, Shanghai Jiao Tong University Affiliated Sixth People’s Hospital, #600, Yishan Road, Shanghai, 200233 China; 2 Department of Surgery, Heilongjiang Provincial Hospital Affiliated to Harbin Institute of Technology, #82, Zhongshan Road, Harbin, 1500036 China

**Keywords:** Adolescent and young patient, Characteristic, Outcome, Hepatocellular carcinoma

## Abstract

**Background:**

The risk of HCC is documented to be age-related. The outcomes of young HCC patients on postoperative prognosis are not well understood. The study aims to compare the characteristic differences between adolescent and young (AYA) and non-AYA HCC patients.

**Methods:**

We performed a retrospective analysis of the clinical and pathological findings and the survival of 243 HCC patients who underwent operations between 2007 and 2018.

**Results:**

The AYA group had a higher AFP level and a higher prevalence of family history of HCC or other cancers than the non-AYA group (*P* < 0.01 and *P* < 0.05). AYA patients had more unfavorable pathological characteristics including bigger lesion size, microvascular invasion, portal vein invasion, and hepatic capsule invasion. They also had a more unfavorable Edmondson grade and less tumor capsule formation (*P* < 0.01). Age was an independent predictor of survival in HCC patients. AYA patients had poorer disease-free and overall survival than non-AYA patients did (*P* < 0.01). Patients under 30 years old had an even poorer disease-free survival than those aged 30–40 (*P* = 0.047).

**Conclusions:**

AYA patients exhibited a higher recurrence rate and disease-related death rate with more unfavorable pathological characteristics. Enhanced follow-up for young HCC patients should be applied.

## Introduction

Hepatocellular carcinoma (HCC) is a worldwide disease [1, 2]. The risk of HCC is documented to be age-related [3, 4]. The adolescent and young adult (AYA), defined as individuals under 40 years old in accordance with the National Comprehensive Cancer Network, is a specific population that has different outcomes as compared to others shown by the AYA Oncology Progress Review Group [5]. Previous studies identified that age difference was associated with the prognosis for such cancers as gastric cancer, breast cancer, and colorectal cancer [6–10]. Few studies have focused on the characteristics and outcomes of AYA HCC patients on postoperative prognosis [11]. This study was designed to compare the outcomes between AYA and non-AYA HCC patients.

## Methods

### Patients

The findings on 243 consecutive patients undergoing curative liver resection for HCC from January 2007 to December 2018 at Shanghai Jiao Tong University Affiliated Sixth People’s Hospital and Heilongjiang Provincial Hospital Affiliated to Harbin Institute of Technology were retrospectively reviewed. The population of this study consisted of 73 patients whose age was under 40 (AYA) and 170 patients whose age was over 40 (non-AYA). The following are the exclusion criteria: (1) age < 20, (2) recurrent HCC, (3) R1 and R2 resection, (4) postoperative death within 30 days, (6) HCC with preoperative treatment, and (7) missing data on important prognostic factors. This study was approved by the ethics committee of Shanghai Jiao Tong University and was conducted in line with the principles of the Declaration of Helsinki. Each patient had a written informed consent.

### Data collection

Patients’ age, sex, HBV infection status, Child-Pugh grading, alanine aminotransferase (ALT), aspartate transaminase (AST), alpha-fetoprotein (AFP), tumor size, tumor number, portal vein invasion, microvascular invasion, satellite nodules, tumor differentiation, tumor encapsulation, family cancer history, and HCC staging [12] were recorded.

### Follow-up

Patients were followed up at a 3-month frequency that was composed of a physical examination and a laboratory test. CT scan was arranged once in 3 months for the first year and then once in 6 months for the second year. The primary end-point was recurrence and death. The primary criterion was the survival time.

### Statistical analysis

Continuous variables were expressed as median and range or mean ± standard deviation. Categorical variables were expressed as number and percentage. The chi-squared test was used for normal data. Univariate analysis was performed using the *χ*^2^ test or Fisher’s exact test for categorical variables. When the data did not normally distribute, the non-parametric Mann-Whitney *U* test was used. The survival was analyzed by the Kaplan-Meier method with the log-rank test. Significant factors in univariate analysis were subjected to multivariate analysis by Cox proportional hazard regression. Data were considered significant for *P* < 0.05. The SPSS 20 statistical software (SPSS, Chicago, IL) was used for analyses.

## Results

### Comparison of the baseline characteristics and pathological outcomes

The AYA group had a higher AFP level and a higher prevalence of family history of HCC or other cancers than the non-AYA group (*P* < 0.01 and *P* < 0.05) (Table 1). No differences were found between the two groups for other laboratory examinations. A much higher proportion of AYA patients had a big tumor size (> 5 cm) (*P* < 0.05), microvascular invasion (*P* < 0.01), portal vein invasion (*P* < 0.01), and hepatic capsule invasion (*P* < 0.01) than that of non-AYA counterparts. The AYA group had a more unfavorable Edmondson grade than the non-AYA group (*P* < 0.01). However, fewer patients in the AYA group had tumor capsule formation (*P* < 0.01) (Table 1).

**Table 1:** Comparison of the baseline characteristics and pathological outcomes between the AYA and non-AYA groups

Characteristics	AYA group (*n* = 73)	Non-AYA group (*n* = 170)	*P* value
Age (years)	36.0 ± 4.4	62.0 ± 6.4	< 0.01
Sex, *n* (%)			
Male	56 (76.7%)	140 (82.4%)	
Female	17 (23.3%)	30 (17.6)	NS
Etiology, *n* (%)			
HBV	60 (82.1%)	141 (83.0%)	
Others	13 (17.8%)	29 (17.0%)	NS
BMI (kg/m^2^)	22.1 ± 1.2	20.6 ± 0.9	NS
AST (U/L)	42 (23–119)	50 (16–269)	NS
ALT (U/L)	62 (22–167)	54 (9–204)	NS
Albumin (g/dL)	4.2 ± 0.4	4.0 ± 0.5	NS
Total bilirubin (mg/dL)	0.7 ± 0.4	0.7 ± 0.4	NS
Platelet (× 10^3^/mm^3^)	199 (40–362)	183 (66–393)	NS
INR	1.1 ± 0.1	1.1 ± 0.1	NS
AFP (ng/dL)	5923 (2–59,403)	3341 (1–59,779)	< 0.01
PIVKA-II	271 (10–500)	238 (11–500)	NS
ICG R15 (%)	8.5 (2.4–41.5)	10.0 (3.7–48.6)	NS
Child-Pugh grade B, *n* (%)	2 (3.0%)	5 (2.9%)	NS
Family history of HCC or other cancers, *n* (%)	15 (20.5%)	20 (11.8%)	< 0.05
Maximum tumor size > 5 cm	42 (57.5%)	76 (44.1)	< 0.05
Multiple tumors	6 (8.2%)	15 (8.8%)	NS
Microvascular invasion	64 (87.7%)	121 (71.2%)	< 0.01
Portal vein invasion	24 (32.9%)	33 (19.4%)	< 0.01
Satellite nodules	12 (16.4%)	31 (18.2%)	NS
Edmondson grade			
I + II	36 (49.3%)	151 (88.8%)	
III + IV	37 (50.7%)	19 (11.2%)	< 0.01
Tumor capsule formation	39 (53.4%)	126 (74.1%)	< 0.01
Hepatic capsule invasion	8 (11.0%)	9 (5.3%)	<0.01
Microvascular tumor emboli	47 (64.4%)	119 (70.0%)	NS
TNM stage			
I + II	25 (34.2%)	68 (40.4%)	
III + IV	48 (65.8%)	102 (60.0%)	NS
Resection margin < 1 cm	36 (49.3%)	77 (45.3%)	NS

*HCC* hepatocellular carcinoma, *NS* not significant

### Survival analysis

**Fig. 1:**
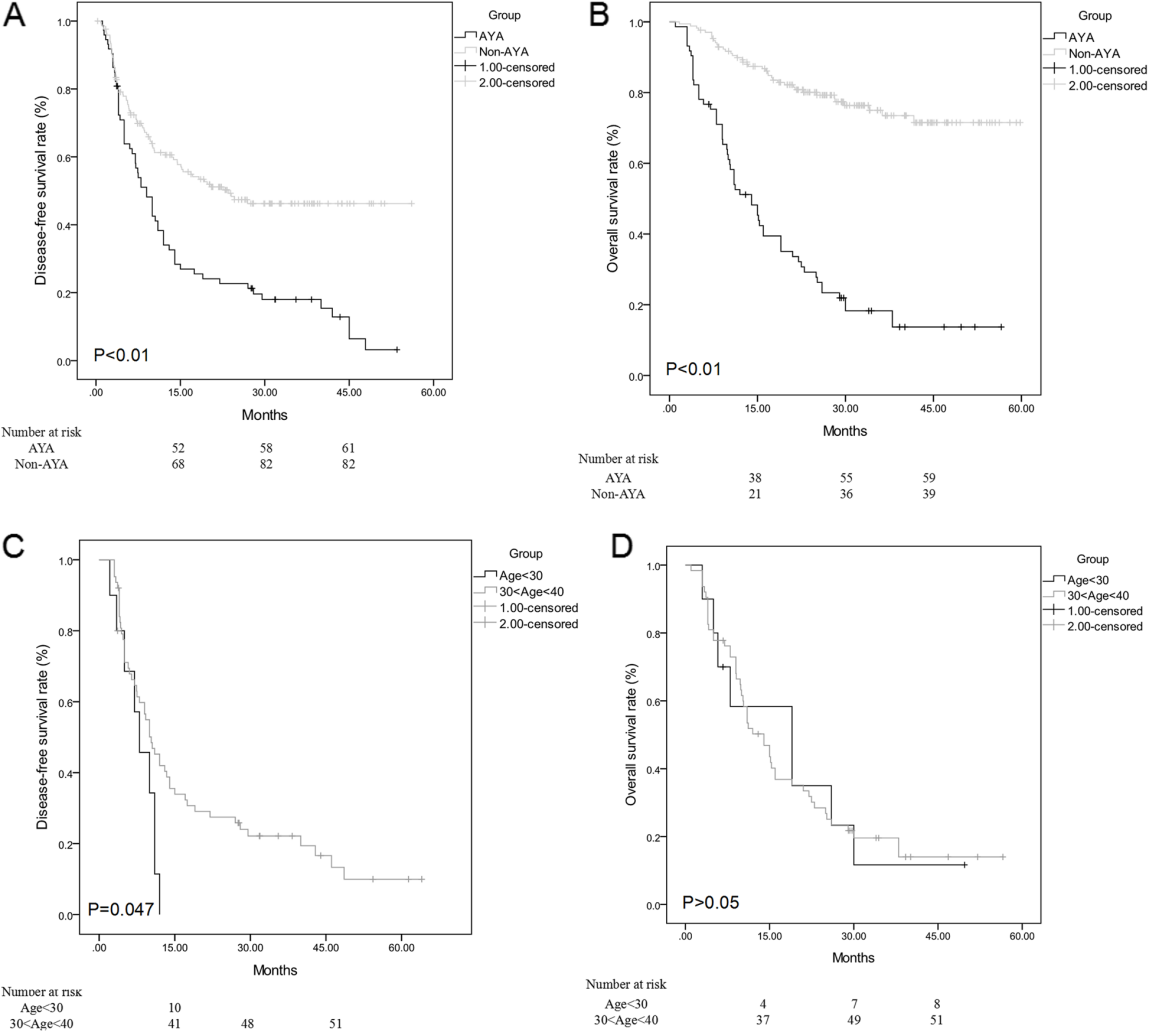
Disease-free survival and overall survival of patients. **A**, **B** AYA patients had poorer disease-free and overall survival than non-AYA patients did (*P* < 0.01). **C**, **D** Patients under thirty had an even poorer disease-free survival than patients aged 30–40 (*P* = 0.047). However, no significance was found in overall survival (*P* > 0.05)

In univariate analysis, age, AFP, maximum tumor size, portal vein invasion, satellite nodules, tumor capsule formation, and TNM stage were strongly associated with disease-free and overall survival in HCC patients. In multivariate analysis, age, maximum tumor size, portal vein invasion, satellite nodules, tumor capsule formation, and TNM stage were strongly associated with disease-free survival while age, AFP, maximum tumor size, portal vein invasion, tumor capsule formation, and TNM stage were strongly associated with overall survival in HCC patients (Table 2). AYA patients had poorer disease-free and overall survival than non-AYA patients did (*P* < 0.01) (Fig. 1A, B and Table 3). In multivariate analysis, maximum tumor size, portal vein invasion, tumor capsule formation, and TNM stage were strongly associated with disease-free survival in both the AYA and non-AYA groups. Maximum tumor size, portal vein invasion, TNM stage, and AFP were strongly associated with overall survival in both groups. Additionally, tumor capsule formation was strongly related to the overall survival in the non-AYA group (Table 4). We further stratified the AYA patients with the cutoff age of 30 years old and performed the survival analysis. As a result, patients under thirty had an even poorer disease-free survival than patients aged 30–40 (*P* = 0.047). But no significance was found in overall survival (*P* > 0.05) (Fig. 1C, D).

**Table 2:** Univariate and multivariate analysis for disease-free survival and overall survival in HCC patients

Characteristics	Univariate analysis				Multivariate analysis			
	Disease-free survival		Overall survival		Disease-free survival		Overall survival	
	HR	95% CI *P* value	HR	95% CI *P* value	HR	95% CI *P* value	HR	95% CI *P* value
Age ≤ 40	2.2	1.2–2.8 < 0.01	2.1	1.4–2.6 < 0.01	1.8	1.1–3.1 < 0.01	2.3	1.3–3.2 < 0.01
AFP ≥ 100 ng/dL	1.2	1.1–2.1 < 0.05	1.6	1.2–2.4 < 0.05			1.9	1.2–3.1 < 0.05
Sex male	0.3	NS	0.4	NS				
HBV-positive	0.5	NS	0.6	NS				
BMI ≥ 23	0.4	NS	0.3	NS				
Family history cancers	0.8	NS	0.7	NS				
Maximum tumor size > 5 cm	1.8	1.2–3.1 < 0.01	1.6	1.2–3.4 < 0.01	2.1	12–4.1 < 0.01	1.8	1.3–3.5 < 0.01
Multiple tumors	0.6	NS	0.4	NS				
Microvascular invasion	0.6	NS	0.8	NS				
Portal vein invasion	2.5	1.2–4.3 < 0.01	2.0	1.3–3.9 < 0.01	3.1	1.9–4.2 *P* < 0.01	2.2	1.3–3.4 *P* < 0.01
Satellite nodules	1.8	1.1–2.7 < 0.05	1.2	1.1–2.9 < 0.05	1.4	1.2–2.9 < 0.05		
Edmondson grade	0.5	NS	0.3	NS				
Tumor capsule formation	0.4	0.1–08 < 0.05	0.6	0.2–0.9 < 0.05	0.5	0.1–0.8 < 0.05	0.7	0.1–0.9 < 0.05
Hepatic capsule invasion	0.3	NS	0.1	NS				
Microvascular tumor emboli	0.4	NS	0.2	NS				
TNM stage	3.1	1.8–4.9 < 0.01	2.2	1.5–3.6 < 0.01	3.0	1.3–4.3 < 0.01	2.1	1.5–3.9 < 0.01
Resection margin < 1 cm	0.7	NS	0.4	NS				

*NS* not significant

**Table 3:** One- and 3-year disease-free and overall survival in the AYA and non-AYA groups

Group	Disease-free survival (%)				Overall survival (%)			
	Median survival time (months)	1 year	3 years	*P* value	Median survival time (months)	1 year	3 years	*P* value
AYA	9.3	34.0	18.0		14.4	51.0	18.0	
Non-AYA	23.3	61.0	46.0	< 0.01	59	89.0	73.0	< 0.01

**Table 4:** Multivariate analysis for disease-free survival and overall survival in the AYA and non-AYA groups

	Disease-free survival			Overall survival		
	HR	95% CI	*P* value	HR	95% CI	*P* value
AYA group						
Maximum tumor size > 5 cm	1.3	1.1–2.4	< 0.01	1.5	1.1–2.7	< 0.01
Portal vein invasion	1.8	1.5–2.3	< 0.01	1.6	1.4–2.4	< 0.01
Tumor capsule formation	0.6	0.3–0.8	< 0.05			
TNM stage	2.1	1.5–3.1	< 0.01	3.1	2.1–5.4	< 0.01
AFP > 100 ng/dL				1.4	1.1–2.5	< 0.01
Non-AYA groups						
Maximum tumor size > 5 cm	1.5	1.2–2.1	< 0.01	1.7	1.2–2.6	< 0.01
Portal vein invasion	2.1	1.3–3.3	< 0.01	2.5	1.4–3.5	< 0.01
Satellite nodules	1.9	1.3–2.7	< 0.05			
Tumor capsule formation	0.5	0.2–0.9	< 0.05	0.7	0.3–0.9	< 0.01
TNM stage	2.7	1.3–4.1	< 0.01	1.8	1.1–3.1	< 0.01
AFP > 100 ng/dL				2.0	1.2–4.1	< 0.05

### Comparison of the pathological outcomes within AYA patients

To identify the cause of even shorter disease-free survival in AYA patients under 30 years old, we conducted a comparison analysis. We found that patients under 30 years old had more unfavorable pathological manifestations including satellite nodules, hepatic capsule invasion, and poor Edmondson grade (*P* < 0.01) (Table 5).

**Table 5:** Pathological outcomes of HCC patients under 40 years old

Variable, *n* (%)	Age < 30 (*n* = 10)	30 < age < 40 (*n* = 63)	*P* value
Satellite nodules	3 (30.0%)	9 (14.3%)	< 0.01
Edmondson grade			
I + II	3 (30.0%)	33 (52.4%)	
III + IV	7 (70.0%)	30 (47.6%)	< 0.01
Hepatic capsule invasion	2 (20.0%)	6 (9.5%)	< 0.01

## Discussion

Among solid tumors in the elderly, the age-related incidence of HCC is found in the population older than 70 years old [13]. HCC is also commonly seen in young patients in places that are endemic to hepatitis B virus such as East Asia [14, 15]. Hepatectomy remains the most effective treatment for HCC despite the unsatisfactory outcomes of HCC associated with portal vein tumor thrombosis [16]. Identification of prognostic risk factors is critical to improve the survival. Considering the small number of AYA HCC samples in many institutions, few studies had focused on the outcomes for the younger patients in particular. This study was designed to identify the factors affecting the survival of AYA HCC patients with a relatively larger case number.

Compared to other patients, AYA represents a unique oncological population in many ways [17]. In some publications, AYA patients were shown to have a poor prognosis because of the advanced stage [18]. However, in other studies, AYA patients shared similar disease-free or overall survival with the older patients, and age itself was an independent factor for prognosis [19, 20]. However, the case numbers enrolled in those studies were small which might lead to statistical bias. In these studies, most AYA patients have a history of hepatitis B with a good liver function that could delay the diagnosis of HCC. Similarly, we found in this study that 82.2% of AYA patients had a positive HBV infection, and 97.0% had a liver function of Child-Pugh A. More importantly, we found that age was an independent predictor of survival in HCC patients. AYA patients had poorer disease-free and overall survival than non-AYA patients did. AYA patients had a higher preoperative AFP level and more unfavorable pathological characteristics including tumor size, microvascular invasion, portal vein invasion, and hepatic capsule invasion. They also had a more unfavorable Edmondson grade and less tumor capsule formation than non-AYA patients did. In addition, we found that AYA patients a higher prevalence of family history of HCC or other cancers than non-AYA patients. It is reasonable to estimate that young HCC patients are likely to have genetic factors contributing to the onset of cancers. A recent study implied that apoptosis-related genes in HCC were associated with the patient’s prognosis [21]. Furthermore, tumor size, vascular invasion, tumor capsule formation, satellite nodules, TNM stage, and AFP were identified as the significant variables both for disease-free and overall survival, which were in line with the previous studies [22–25]. Zhao et al. found that BMI did not affect the patient’s long-term surgical outcomes concerned to overall and disease-free survival [26], and in line with which, we did not find the difference in BMI between these two groups and its impact on patient’s survival. In agreement with Saito et al. [25], we found that the larger tumor size was associated with unfavorable outcomes. In addition, another study reported that the very early recurrence nomogram contained microvascular invasion, macrovascular invasion, and CA199 level [27]. In this study, we further stratified the AYA patients with the cutoff age of 30 years old and performed the survival analysis again. Interestingly, we found that patients under thirty had an even poorer disease-free survival than patients aged 30–40. To investigate the cause of this finding, we conducted a comparison analysis. We found that patients under thirty had more unfavorable pathological manifestations including satellite nodules, hepatic capsule invasion, and poor Edmondson grade. This study had some limitations concerning its retrospective nature. Though there were limitations in this study, the differences between the groups were remarkable.

## Conclusions

In conclusion, AYA patients exhibited a higher risk of recurrence and disease-related death with more unfavorable pathological characteristics. Enhanced follow-up for those chronic hepatitis B AYA carriers should be applied. Adjuvant sorafenib therapy after resection in HCC patients is suggested since it prolongs the overall and disease-free survival and reduces the recurrence rate without intolerable side effects [28].

## Data Availability

The datasets used and analyzed during the current study are available from the corresponding authors on reasonable request.

## Abbreviations

AYA: Adolescent and young adult
HCC: Hepatocellular carcinoma
ALT: Alanine aminotransferase
AST: Aspartate transaminase
AFP: Alpha-fetoprotein
